# Epigenetic insights into Fragile X Syndrome

**DOI:** 10.3389/fcell.2024.1432444

**Published:** 2024-08-16

**Authors:** Liangqun Xie, Huiying Li, MengLiang Xiao, Ningjing Chen, Xiaoxiao Zang, Yingying Liu, Hong Ye, Chaogang Tang

**Affiliations:** ^1^ The Fifth Affiliated Hospital of Sun Yat-sen University, Zhuhai, China; ^2^ Department of Obstetrics and Gynecology, The First College of Clinical Medical Science, Yichang Central People’s Hospital, Three Gorges University, Yichang, Hubei, China

**Keywords:** Fragile X Syndrome, FMR1 silencing, methylation, acetylation, chromatin remodeling, non-coding RNA

## Abstract

Fragile X Syndrome (FXS) is a genetic neurodevelopmental disorder closely associated with intellectual disability and autism spectrum disorders. The core of the disease lies in the abnormal expansion of the CGG trinucleotide repeat sequence at the 5′end of the FMR1 gene. When the repetition exceeds 200 times, it causes the silencing of the FMR1 gene, leading to the absence of the encoded Fragile X mental retardation protein 1 (FMRP). Although the detailed mechanism by which the CGG repeat expansion triggers gene silencing is yet to be fully elucidated, it is known that this process does not alter the promoter region or the coding sequence of the FMR1 gene. This discovery provides a scientific basis for the potential reversal of FMR1 gene silencing through interventional approaches, thereby improving the symptoms of FXS. Epigenetics, a mechanism of genetic regulation that does not depend on changes in the DNA sequence, has become a new focus in FXS research by modulating gene expression in a reversible manner. The latest progress in molecular genetics has revealed that epigenetics plays a key role in the pathogenesis and pathophysiological processes of FXS. This article compiles the existing research findings on the role of epigenetics in Fragile X Syndrome (FXS) with the aim of deepening the understanding of the pathogenesis of FXS to identify potential targets for new therapeutic strategies.

## 1 Introduction

Fragile X Syndrome (FXS) is a common cause of hereditary intellectual disability (ID) and autism spectrum disorders (Budimirovic et al., 2020; Salcedo-Arellano et al., 2020). Its clinical manifestations include characteristic facial features, macroorchidism, learning disabilities, cognitive impairments, behavioral disorders, and autism spectrum disorders, as well as epileptic seizures (Salcedo-Arellano et al., 2020; Stone et al., 2023; Svalina et al., 2023). The underlying genetic cause of FXS is attributed to the abnormal expansion of the CGG repeat sequence located within the 5′-untranslated region of the Fragile X mental retardation 1 gene (FMR1). When this CGG trinucleotide repeat surpasses 200 units, it triggers the silencing of the FMR1 gene, resulting in a deficiency of the Fragile X mental retardation protein 1 (FMRP) (Ranjan et al., 2023; Stone et al., 2023; Svalina et al., 2023).

**FIGURE 1:**
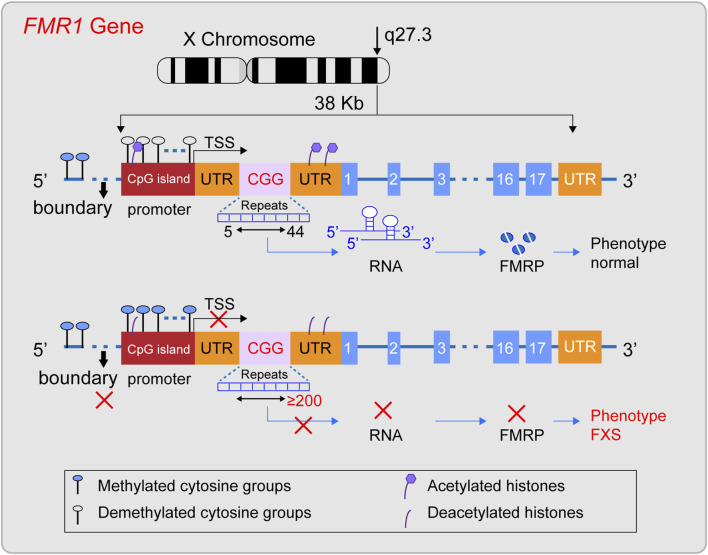
Diagram illustrating the structural characteristics of a normal FMR1 gene and one with a full mutation. The FMR1 gene, situated on the X chromosome’s q27.3 region, typically features a CGG repeat sequence between 5 and 44 times. In this state, the gene’s methylation boundaries are well-defined, the promoter remains free of methylation, and histones are acetylated, facilitating the gene’s transcription into mRNA and the production of the FMRP protein, thus preserving a normal phenotype. However, when the CGG repeat count surpasses 200, a full mutation occurs, erasing the methylation boundaries, methylating the promoter region, and deacetylating histones. This sequence of events halts the transcription of the FMR1 gene into mRNA, leading to the absence of the FMRP protein and potentially causing Fragile X Syndrome (FXS).

The FMR1 gene was successfully cloned in 1991 (Verkerk et al., 1991) and is located in the Xq27.3 region (Nobile et al., 2021; Tabolacci et al., 2022). The gene is approximately 38 kilobases long and includes, from the 5′end to the 3′end, the promoter region, the 5′untranslated region (5′UTR), exons and introns, and the 3′untranslated region (3′UTR) (Zafarullah et al., 2020) (Figure 1). The promoter region lacks a typical TATA box and contains a CpG island composed of about 56 CpG dinucleotides, two GC boxes, and three Inr-like sequences (Nobile et al., 2021; Ciobanu et al., 2023). The 5′UTR is rich in CGG repeat sequences, which are located about 130 nucleotides from the Inr-like sequence of the promoter and approximately 50 nucleotides from the transcription start site (Nobile et al., 2021). The FMR1 gene includes 17 exons and 16 introns, with the first exon marking the beginning of the open reading frame (ORF) (Verkerk et al., 1991; Barasoain et al., 2016). Under normal conditions, the CGG repeat sequence in the 5′UTR of the FMR1 gene repeats between 5 and 44 times. Upstream of the CGG repeat sequence, there is a unique DNA methylation boundary about 650–800 nucleotides from exon 1. The promoter’s upstream region is methylated, while the promoter and the downstream of the FMR1 gene remain unmethylated. Meanwhile, the histones H3 and H4 of the FMR1 gene are acetylated (Coffee et al., 1999; Naumann et al., 2010), maintaining the gene’s normal transcriptional and translational state. Its ORF can be transcribed into a 4.4 kb precursor mRNA (Nobile et al., 2021), and through alternative splicing, it specifically expresses different isoforms of FMRP protein in different tissue cells or developmental stages (Ferder et al., 2022). FMRP is an important RNA-binding protein that plays a key role in various biological processes, including synaptic plasticity, neuronal development, and cellular signaling (Darnell et al., 2011; Casingal et al., 2020; Monday et al., 2022; Martín et al., 2023; Yu et al., 2023). It is crucial for maintaining the normal function of the nervous system, especially in the brain, where it represses about 4% of the brain’s mRNAs (Rodriguez et al., 2020), When the CGG repeat sequence ranges from 45 to 54, it is considered a gray zone. Between 55 and 200, it is referred to as a premutation, which may be associated with the development of Fragile X-associated Primary Ovarian Insufficiency (FXPOI) and Fragile X-associated Tremor/Ataxia Syndrome (FXTAS). The underlying pathogenic mechanism might involve the overexpression of the FMR1 gene’s mRNA, leading to a toxic effect (Huang et al., 2019). In FXS, when the (CGG) n repeat sequence in the 5′untranslated region (5′UTR) expands to 200 or more repeats, this is known as a full mutation (FM). Against the backdrop of a FM, while the promoter sequence and the open reading frame (ORF) of the FMR1 gene remain unchanged, the methylation boundary region of the FMR1 gene may vanish. This leads to a state of heightened methylation across the region spanning from the promoter to intron 1 of the FMR1 gene. Additionally, histones H3 and H4 associated with the FMR1 gene undergo deacetylation (Suardi and Haddad, 2020; Nobile et al., 2021). Consequently, the FMR1 gene lapses into a state of transcriptional silencing, which in turn prevents the normal expression of the FMRP protein.

Although the expansion of the CGG repeat sequence is a known cause of FMR1 gene silencing, the specific molecular mechanisms are not yet clear. Currently, it is known that the silencing of the FMR1 gene is associated with epigenetic changes such as DNA methylation and histone deacetylation. Moreover, research indicates that the gene may have acquired additional inhibitory chromatin modifications, such as H3K9me2 and H3K27me3, as well as chromatin compaction (Usdin and Kumari, 2015). In addition, clinical observations have noted that a small number of full mutation carriers without changes in epigenetic markers have normal intelligence (Smeets et al., 1995), which further emphasizes the importance of epigenetics in the process of FMR1 gene silencing.

Epigenetics refers to heritable changes in cell phenotype that do not involve alterations to the DNA sequence itself, including but not limited to methylation, histone modification, chromatin remodeling, and non-coding RNA regulation (Peixoto et al., 2020; Dai et al., 2021). In recent years, researchers have also begun to focus on other emerging epigenetic phenomena, such as the formation of R-loops and the three-dimensional(3D) genome regulation (Kim and Wang, 2021; Glaser and Mundlos, 2022). These mechanisms play a crucial role in determining cellular fate, working independently or in concert to regulate gene expression in a reversible manner (Kim and Wang, 2021; Glaser and Mundlos, 2022; Madrigal et al., 2023). Epigenetic mechanisms have been proven to be closely related to the occurrence and development of a variety of diseases, such as FXS (Poeta et al., 2020). These mechanisms not only help to understand the pathogenesis of diseases, but they themselves may also become potential therapeutic targets for these diseases.

This article presents a comprehensive review of the current epigenetic research findings within the domain of Fragile X Syndrome (FXS). It encompasses pivotal mechanisms such as DNA methylation, histone acetylation, chromatin remodeling, and the role of non-coding RNAs along with other emerging epigenetic phenomena. The objective is to shed light on the intricate molecular mechanisms underlying the silencing of the FMR1 gene and to facilitate the translation of epigenetic theories from the realm of basic science to practical clinical applications.

## 2 Methylation changes and FXS

Methylation changes are a key phenomenon in epigenetics, which refers to the genetic changes caused by the methylation or demethylation of targets by methylation or demethylation enzymes, without altering the DNA sequence. These changes are mainly divided into three categories: DNA, RNA, and protein methylation changes (Dai et al., 2021).

### 2.1 DNA methylation and FXS

DNA methylation alterations refer to the addition or removal of methyl groups at specific bases within the DNA molecule, such as the N6 position of adenine, the N7 position of guanine, and the C5 position of cytosine (Dai et al., 2021). In prokaryotes, methylation predominantly occurs at restriction enzyme sites, serving a protective role for the DNA. In contrast, in eukaryotes, methylation is predominantly found at cytosines within CpG islands, resulting in the formation of 5′-methylcytosine (5′-mC) structures (Mattei et al., 2022). Approximately 70% of promoters in the human genome contain CpG islands, including the FMR1 gene (Nobile et al., 2021; Fan et al., 2023), highlighting the ubiquity of DNA methylation in gene regulation.

DNA methylation is orchestrated by a suite of enzymes, including DNA methyltransferases (DNMTs) such as DNMT1, DNMT3A, DNMT3B, and the DNMT3L, as well as the demethylation TET family enzymes (Chen and Zhang, 2020; Acharjee et al., 2023). DNMT1 predominantly perpetuates established methylation patterns, whereas DNMT3A and DNMT3B are instrumental in laying down new methylation profiles during embryogenesis. Although DNMT3L lacks enzymatic activity as a methyltransferase, it plays a supportive role in the function of DNMT3A and DNMT3B. The TET family of enzymes initiates the demethylation process by converting 5-methylcytosine (5 mC) to 5-hydroxymethylcytosine (5 hmC), which can then undergo further oxidation to 5-formylcytosine and 5-carboxylcytosine (Charlton et al., 2020; Feng et al., 2024). This process reveals the dynamics and reversibility of DNA methylation modification.

DNA methylation plays a key role in the suppression of gene transcription. In the upstream region of a gene, the differential methylation of CpG islands can attract specific proteins to achieve gene silencing. Downstream of the gene, DNA methylation in the imprinting control region (ICR) suppresses gene expression by regulating gene transcription (Sergeeva et al., 2023). These regulatory effects are closely connected with other epigenetic modifications such as histone methylation and acetylation (Hashimoto et al., 2010; Cusack et al., 2020; Tibben and Rothbart, 2024). For histone methylation, whole-genome DNA methylation profiling studies have shown that DNA methylation is associated with specific histone methylation patterns, especially H3K4me0 and H3K9 methylation, with H3K9 methylation potentially being a prerequisite for the occurrence of DNA methylation (Hashimoto et al., 2010). DNA methylation may also promote the process of lysine methylation (Cusack et al., 2020). As for histone acetylation, many studies have confirmed that methylated CpG sites can attract proteins containing a methyl-CpG binding domain (MBD). These proteins further recruit histone deacetylase complexes, leading to histone deacetylation, which results in gene heterochromatinization, etc (Desai et al., 2015; Leighton et al., 2023). That is, these interactive relationships affect the binding capacity of transcription factors to DNA, thereby regulating the conformation of chromatin and the transcriptional activity of genes (Kreibich and Krebs, 2023). These findings highlight the complexity of epigenetic regulation and emphasize the importance of considering these interrelationships in disease research.

DNA methylation is a crucial player in the pathogenesis of FXS. The prevailing view among researchers is that the expansion of the CGG repeat sequence in the FMR1 gene beyond 200 repeats leads to methylation of the CpG island in the promoter region and the erosion of the methylation boundary upstream of the promoter, which are the primary triggers for the silencing of the FMR1 gene (Naumann et al., 2010; Naumann et al., 2014). This hypothesis is reinforced by numerous studies, including cases of full mutation carriers with normal cognitive function (Smeets et al., 1995), research demonstrating a positive correlation between the extent of DNA methylation and the severity of the FXS phenotype (Baker et al., 2019; Khoodoruth et al., 2024), and some therapeutic research, including case studies that demonstrate treatment with 5-azacitidine can block DNA methylation and reactivate the FMR1 gene (Tabolacci et al., 2016a), as well as targeted DNA demethylation using the dCas9-Tet1 system can restore the activity of the FMR1 gene to a certain extent (Liu et al., 2018).

However, the precise mechanism by which the expansion of CGG repeats leads to hypermethylation of FXS CpG cytosines is not yet fully understood. The extension of the CGG repeat sequence beyond the methylation boundary and the potential insertion of foreign DNA are hypothesized to be contributing factors (Naumann et al., 2014). As research progresses, emerging findings are challenging the notion that DNA methylation is the sole primary factor in the silencing of the FMR1 gene. Firstly, the inactivation of the FMR1 gene is associated not only with DNA demethylation but also with increased levels of deacetylation of histones H3 and H4 (Pietrobono et al., 2005; Li et al., 2018), suggesting that other epigenetic modifications, in addition to DNA methylation, play a critical role in the pathogenesis of FXS. Secondly, numerous experiments have shown that histone deacetylase (HDAC) inhibitors can activate the FMR1 gene and ameliorate FXS symptoms, even without altering DNA methylation activity (Biacsi et al., 2008; Tabolacci et al., 2008). Some researchers have posited that the silencing of FMR1 may be triggered by chromatin modification changes due to histone deacetylation, which may occur prior to DNA methylation (Eiges et al., 2007; Warren, 2007), further underscoring the complexity of epigenetic regulation. Additionally, 5-azacitidine (5-AzadC) cannot reactivate the silenced the FMR1 gene in post-mitotic neurons and can only exert its effects during the DNA replication process (Kumari et al., 2020), indicating that DNA methylation may not be the sole factor in the pathogenesis of FXS and that the role of other epigenetic modifications should not be disregarded. These research outcomes underscore the importance of considering a broader spectrum of epigenetic regulatory mechanisms, rather than focusing solely on DNA methylation, in the development of therapeutic strategies for FXS.

### 2.2 RNA methylation and FXS

RNA methylation is a form of epigenetic modification found in a variety of RNA molecules, including messenger RNA (mRNA), transfer RNA (tRNA), ribosomal RNA, and small nuclear RNA. Over 100 types of RNA methylation are known, with N6-methyladenosine (m6A) and 5-methylcytosine (m5C) being the most common (Yang et al., 2021). m6A methylation is primarily catalyzed by the METTL3/14 methyltransferase complex and the WTAP protein, while the demethylation process is mainly carried out by the FTO and ALKBH5 enzymes (An and Duan, 2022). The m5C methylation is mediated by members of the NOP2/Sun RNA methyltransferase family and the DNMT2 family of DNA methyltransferases, and the demethylation is performed by the TET family of enzymes (Balachander et al., 2023). m6A and m5C meticulously control gene expression by precisely regulating key steps such as RNA splicing, localization, stability, nuclear export, degradation, transcription, and translation, thereby causing physiological and pathological changes in cells and having a profound impact on cellular function and the development of diseases.

The m6A and m5C methylation modifications have been established as pivotal factors in the etiology of FXS. With respect to m6A modification, research indicates that the FMRP predominantly targets mRNAs that have been marked with m6A. For example, the sites where FMRP binds to mRNAs correlate closely with the locations of m6A modifications on these mRNAs (Chen et al., 2023); the neurodevelopmental abnormalities caused by FMRP deficiency share similarities with the neurodevelopmental phenotypes resulting from abnormal m6A modification (Edens et al., 2019); in the mouse cortex, lack of FMRP can affect the m6A landscape (Zhang et al., 2018). Furthermore, FMRP regulates the stability of m6A-marked mRNAs through interaction with the m6A reader protein YTHDF2, and promotes the export of m6A-marked RNA targets from the nucleus to the cytoplasm, affecting the differentiation of neural progenitor cells (Edens et al., 2019). Regarding m5C modification, an increasing number of studies suggest that FMRP may act as an emerging m5C reader protein, participating in the regulation of the establishment and removal of m5C methylation modifications (Yang et al., 2022; Chen et al., 2023). Nevertheless, the specific mechanisms of RNA methylation in FXS still require further research to fully elucidate its biological significance.

### 2.3 Protein methylation and FXS

Protein methylation is divided into histone and non-histone methylation. Histone methylation primarily occurs on the lysine and arginine residues of histones and is jointly regulated by methyltransferases such as PRMT (Protein Arginine Methyltransferase) and KMT (Lysine Methyltransferase), as well as demethylases like LSD1 (Lysine-Specific Demethylase 1) to maintain balance (Gong and Miller, 2019; Dai et al., 2021). The histones methylation, in addition to its close ties with DNA methylation, also engages in a dynamic, antagonistic interplay with histone acetylation (Zhang et al., 2021). Studies have shown that many residues of histone methylation can also undergo acetylation. For instance, the acetylation at the H3K9 site must be removed before methylation can occur (Monte-Serrano et al., 2023). This fine regulatory mechanism is crucial for the regulation of gene expression in cells. In the cell, many histone methylation is associated with gene silencing regions, such as H3K9, H3K27, and H4K20 (Zhang et al., 2021). These specific histone modifications typically mark a variety of transcriptional repression sites and areas where heterochromatin is formed (Gong and Miller, 2019; Zhang et al., 2021; Monte-Serrano et al., 2023). The degree of methylation at these sites determines the openness or closure of chromatin, thereby finely regulating the activity of gene expression. Specifically, the tri-methylation of H3K9 (H3K9me3) is generally closely related to the formation of heterochromatin and gene silencing (Methot et al., 2021; Padeken et al., 2022), while the tri-methylation of H3K27 (H3K27me3) is associated with the suppression of gene expression, playing a crucial role in the silencing of specific genes during the developmental process of an organism (Nichol et al., 2016; Laugesen et al., 2019). Non-histone methylation typically targets lysine and arginine residues on proteins engaged in signal transduction, playing a role in diverse signaling pathways like MAPK, WNT, and BMP (Dai et al., 2021; Chen et al., 2024). This methylation fine-tunes protein activity by influencing these pathways and directs the translation, localization, and signal transduction processes of proteins.

In research on FXS, it has been found that the silenced FMR1 gene region is enriched with various histone methylation marks associated with transcriptional silencing, such as H3K9me2, H3K27me3, H3K9me3, and H4K20me3 (Kumari and Usdin, 2010; Basalingappa, 2020). The common characteristics of these modifications give the FMR1 locus heterochromatic properties, thereby suppressing the transcription of the FMR1 gene. Experiments have shown that removing these modifications can effectively reverse the symptoms of FXS (Tabolacci et al., 2016b; Kumari and Usdin, 2016; Kumari et al., 2020; Kumari et al., 2024), further demonstrating the importance of histone modifications in the pathogenesis of FXS. In contrast, the study of non-histone modifications in FXS is not as in-depth, but it is known that abnormalities in the WNT, BMP, and MAPK signaling pathways are associated with FXS (Casingal et al., 2020; Salcedo-Arellano et al., 2021; Song and Broadie, 2022).

## 3 Histone acetylation and FXS

Histone acetylation is crucial for regulating gene transcription, being notably more prevalent in open chromatin regions compared to the more condensed heterochromatic regions. This dynamic process is intricately linked to DNA accessibility: DNA is compacted into a chromatin structure, with nucleosomes as its basic units, which include four types of histone proteins: H2A, H2B, H3, and H4 (Ahmad et al., 2022). Specific regions of these histone proteins can undergo modifications that regulate the tight binding of nucleosomes to DNA, thereby controlling gene transcription (Ahmad et al., 2022). The acetylation and deacetylation of lysine residues on the histone tails are among the most common modifications, jointly regulated by histone acetyltransferases (HATs) and histone deacetylases (HDACs) (Kumar et al., 2022; Shvedunova and Akhtar, 2022). When histones are acetylated, it reduces the electrostatic attraction between histones and DNA, leading to a more “open” chromatin structure that facilitates gene transcription; conversely, deacetylation results in a more compact chromatin structure, inhibiting gene transcription (Kumar et al., 2022). Furthermore, histone acetylation is closely related to DNA methylation and histone methylation (see the previous text for details). They are interwoven with each other, forming a complex epigenetic network that plays a regulatory role in maintaining the normal physiological functions of cells and in the occurrence and progression of many diseases.

In research on FXS, the discovery of hypoacetylated FMR1 genes has provided new insights into the mechanisms underlying the gene’s silencing. Numerous studies have shown that the FMR1 gene in its silenced state has a higher level of histone deacetylation compared to when it is actively transcribed (Pietrobono et al., 2005; Usdin and Kumari, 2015; Reches, 2019; Shitik et al., 2020). Further investigation has shown that within the scope of FXS, the FMR1 gene’s methylated CpG sites engage with methylation-binding proteins (MBDs), These interactions subsequently draw in histone deacetylases, triggering histone deacetylation. This process culminates in a condensed chromatin architecture that precipitates gene silencing (Shitik et al., 2020; Dhar et al., 2021). These findings have led researchers to hypothesize that the long CGG repeats at FMR1 gene may guide local histone deacetylation through DNA methylation, thereby contributing to gene silencing. In cellular therapy experiments targeting FXS, the use of the demethylating agent 5-azacitidine (5-azadC) alone significantly activates FMR1 expression in FXS cell lines. In contrast, the use of histone deacetylase inhibitors such as TSA, romidepsin, and vorinostat alone has limited or insignificant effects on the activation of FMR1 (Dolskiy et al., 2017). This result suggests that DNA methylation may play a more critical role in the process of gene inactivation, further supporting the theory that DNA methylation contributes to gene silencing by guiding the deacetylation of local histones.

However, when treating FXS models with the DNA methyltransferase inhibitor 5-aza-dC, it was found that it could only partially reactivate the gene, and this effect required the process of DNA replication to be realized (Kumari et al., 2020; Berry-Kravis et al., 2021). The use of histone deacetylase (HDAC) inhibitors such as sodium valproate and splitomicin has been shown to ameliorate some symptoms in FXS (Tabolacci et al., 2008; Berry-Kravis et al., 2021). In FXS lymphoblastoid cells, sodium valproate has been demonstrated to moderately affect histone modifications at the FMR1 locus, but it does not impact DNA methylation nor significantly influence transcriptional reactivation (Warren, 2007; Tabolacci et al., 2008). This has led some researchers to propose that histone deacetylation may be a key initiating factor in the silencing of the FMR1 gene. Nevertheless, it is currently unclear whether DNA methylation or histone deacetylation plays a decisive role in the silencing of the FMR1 gene, and this point remains to be further investigated.

## 4 Chromatin remodeling and FXS

Chromatin remodeling is an epigenetic process that modulates gene transcription by adjusting the architecture of chromatin through modifications in nucleosome composition and positioning. This is facilitated by chromatin remodeling complexes, which possess ATPase activity and utilize the energy from ATP hydrolysis to enact these changes (Reyes et al., 2021). These factors are categorized into four main classes based on the sequence and structural characteristics of their ATPase subunits: SWI/SNF, ISWI, INO80, and CHD (Clapier and Cairns, 2009; Reyes et al., 2021), each with distinct non-redundant roles within the cell. The SWI/SNF class, which includes complexes such as canonical BAF (cBAF), polybromo-associated BAF (PBAF), and noncanonical BAF (ncBAF), primarily functions to disrupt nucleosome order, promote their disassembly and repositioning, and enhance DNA accessibility (Centore et al., 2020). ISWI exists in complex forms like NURF, CHRAC, and ACF, with subunits that participate in nucleosome maturation and arrangement, maintaining DNA stability (Barisic et al., 2019; Li et al., 2021). The INO80 class, encompassing INO80 and SWR1 complexes, regulates nucleosome sliding and facilitates the exchange of histone variants, impacting DNA status and gene expression (Willhoft and Wigley, 2020; Zhang et al., 2023). The CHD class, which includes CHD1 subunits and the NuRD family, modulates gene transcription by regulating nucleosome assembly, sliding, and disassembly (Muhammad et al., 2023). Chromatin remodeling factors are crucial for cellular function, precisely regulating chromatin structure and influencing cell development, differentiation, and adaptability to the environment.

In the pathogenesis of FXS, chromatin remodeling plays a crucial role. On one hand, these factors may be the key drivers of FMR1 gene silencing. In the context of FXS, the silencing of the FMR1 gene coexists with chromatin remodeling phenomena (Shitik et al., 2020). The latest research reveals that the methylated FMR1 gene can recruit MeCP2 protein, and there is a unique interaction between MeCP2 and specific subunits of the SWI/SNF complex. This interaction may cause a change in the topological structure of the FMR1 gene, thereby triggering the gene’s silencing (Harikrishnan et al., 2005). On the other hand, the imbalance in the expression of chromatin remodeling factors is considered a key factor in the formation of abnormal neurological phenotypes in FXS. In FXS research, the absence of FMRP protein is associated with significant changes in the expression of subunits of chromatin remodeling factors such as the Arid1 family, CHD family, and Smarca family (Korb et al., 2017; Richter and Zhao, 2021), which are closely related to neurodevelopment (Moffat et al., 2022; Wischhof et al., 2022; Liu et al., 2023; Loblein et al., 2023). The latest research using organoids to simulate FXS phenotypes further confirms that the absence of FMRP protein may lead to an increase in the level of CHD remodeling factor subunit CHD2, causing an imbalance in genomic transcription, and ultimately leading to abnormal neurodevelopment (Kang et al., 2021). In summary, chromatin remodeling is central to the pathogenesis of FXS. By thoroughly investigating the specific roles of chromatin remodeling factors in FXS, we can gain a more profound understanding of the disease’s development and progression, laying a solid scientific foundation for the development of targeted therapeutic strategies.

## 5 Non-coding RNA and FXS

Non-coding RNAs (ncRNAs) are a class of RNAs transcribed from genomic DNA that do not encode proteins but make up the vast majority of RNAs produced by the human genome transcription (Bhatti et al., 2021; Yan and Bu, 2021). ncRNAs are divided into two main categories: small non-coding RNAs, which are less than 200 nucleotides in length, and long non-coding RNAs, which are longer than 200 nucleotides. siRNAs and miRNAs belong to the category of small non-coding RNAs. Although they originate from different sources, their primary functions are similar—they bind to the complementary sequences of target mRNAs, activating the RNA-induced silencing complex (RISC), which promotes the degradation of the target mRNAs (Vos et al., 2019; Yan and Bu, 2021). lncRNAs and circRNAs are types of long non-coding RNAs. lncRNAs have a linear structure, while circRNAs form closed circular structures. They can be transcribed in either direction from various regions of protein-coding genes, including exons, introns, and 5′/3′untranslated regions (Panni et al., 2020). These RNA molecules can fold into complex secondary structures and serve diverse biological functions. For example, they can act as “sponges” for miRNAs, preventing them from degrading target mRNAs; they can regulate the binding of transcription factors to promoters, affecting gene expression; and they can also act as “molecular scaffolds”, modulating protein interactions and their downstream signaling pathways (Panni et al., 2020; Yan and Bu, 2021). Overall, ncRNAs play a crucial role in regulating gene expression, constructing cellular structures, modulating protein functions, and participating in numerous biological processes.

**FIGURE 2:**
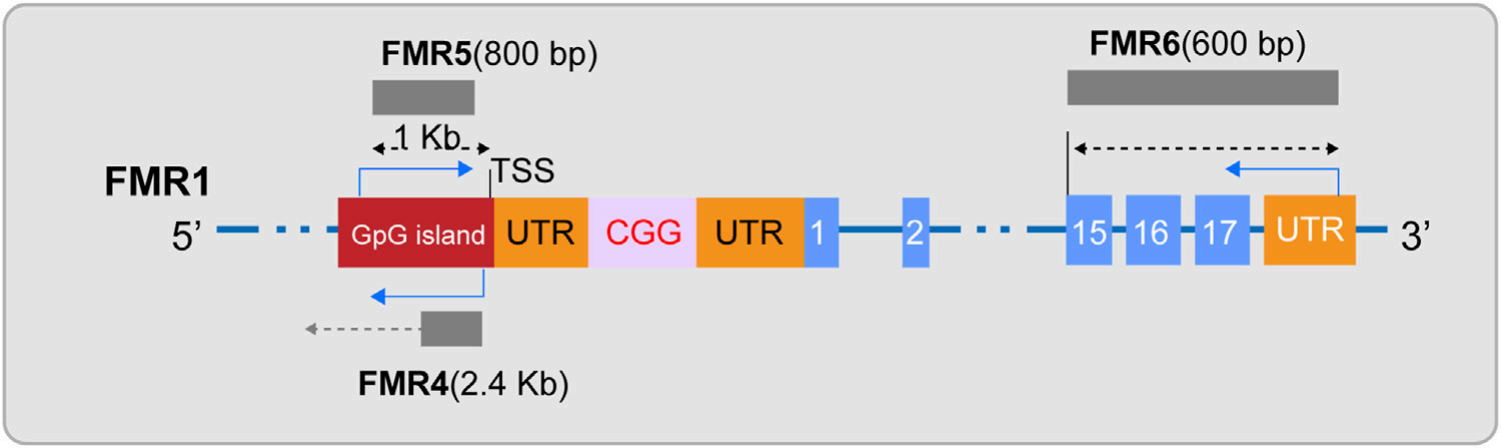
Diagram of lncRNAs FMR4, FMR5, and FMR6. FMR4, a long non-coding RNA (lncRNA), is transcribed by RNA polymerase II and is located upstream of the FMR1 gene in an antisense orientation. FMR5, with its 5′end approximately 1 kilobase upstream of the FMR1 transcription start site (TSS), is an unspliced transcript of approximately 800 nucleotides in length, guided by the sense strand, and overlaps with the promoter region of FMR1. FMR6 is a spliced long non-coding RNA, an approximately 600-nucleotide long antisense transcript that is fully complementary to the 3′region of FMR1. It initiates at the 3′untranslated region (UTR) and terminates within the 15th exon of FMR1.

In the field of FXS research, ncRNAs has become a key focus in exploring the disease’s pathogenesis. Initially, siRNA and miRNA, as small RNA molecules, provide a hypothetical model for RNA-based silencing of the FMR1 gene. Specifically, research indicates that the stable hairpin structure of pre-mutation CGG repeat sequences can be processed by Dicer to produce small RNAs (Handa et al., 2003), and considering the role of siRNA in the formation of heterochromatin (Verdel et al., 2004), researchers have proposed an innovative hypothetical model: the hairpin structured RNA formed after transcription of the fully mutated FMR1 gene can be processed into siRNAs. The deposition of these siRNAs in the expanded CGG repeat region can effectively recruit the RITS complex and further attract key epigenetic effectors, such as histone methyltransferases and DNA methyltransferases, thereby triggering the epigenetic silencing of the FMR1 gene (Jin et al., 2004; Zhou et al., 2019). Secondly, miRNAs may have subtle interactions with FMRP in the pathogenesis of FXS. Extensive research in FXS models has observed that FMRP can control neuronal development by regulating miRNA expression (Liu et al., 2015; Men et al., 2020; Zhang et al., 2020). Conversely, the abnormal expression of specific miRNAs can disrupt FMRP function, weakening its role as a translational repressor in regulating axonal growth and synaptic plasticity, thus inducing FXS-related phenotypes (Wu et al., 2019; Zhang et al., 2019; Lannom et al., 2021). Additionally, lncRNAs are also considered participants in the pathogenesis of FXS (Figure 2). Studies have shown that FMR4 plays an important role in neurodevelopment (Zhao et al., 2020; Barros et al., 2021), but FMR4 is not transcribed in FXS alleles (Shitik et al., 2020; Nobile et al., 2021). Both FMR5 and FMR6 can influence the expression and function of the FMR1 gene (Rosario and Anderson, 2020; Shitik et al., 2020), yet their expression or function is significantly altered in FXS (Huang et al., 2019). Furthermore, circular RNAs (circRNAs) have been confirmed to interact with the FMR1 gene (Liao et al., 2022; Zhong et al., 2023), suggesting a potential link to the pathological processes of FXS. Overall, non-coding RNAs play a multifaceted and critical role in the pathogenesis of FXS. The regulatory effects of these molecules offer new potential targets for the treatment of FXS.

## 6 FXS and other epigenetics

As research continues to deepen, R-loops and 3D genome regulation have become hot topics in the field of epigenetics. R-loops are a three-stranded nucleic acid structure composed of an RNA-DNA hybrid and a displaced single-strand of DNA; they are typically concentrated in the transcription start and termination regions of genes, especially in guanine-rich areas (Niehrs and Luke, 2020). R-loops play the role of dynamic regulators in gene transcription regulation. On one hand, the formation and stability of R-loops are finely regulated by various genetic activities involving numerous epigenetic mechanisms. For instance, DNA methylation can inhibit the formation of R-loops, while N6-methyladenosine (m6A) modification helps stabilize them. In addition, long non-coding RNAs (lncRNAs) and circular RNAs (circRNAs) are also involved in the formation of R-loops (Ginno et al., 2012; Niehrs and Luke, 2020). On the other hand, the presence and stability of R-loops can also cause multiple changes in gene activity. If R-loops are not correctly cleared or shielded, they may cause DNA breaks and activate DNA repair mechanisms. At the same time, R-loops can also alter the structure of chromatin, affecting the methylation modification of certain regions. Therefore, R-loops are a complex epigenetic phenomenon with profound effects on gene activity. 3D genome regulation focuses on how the spatial structure of the genome affects gene expression and regulation. Research in this field is based on 3C technology and its derivatives, revealing multi-level regulatory mechanisms from chromosome territories to chromatin loops. Chromosome territories demonstrate the specific distribution patterns of chromosomes within the cell nucleus, where gene-rich regions gather to form active expression domains, while regions with fewer genes are concentrated within the chromosomes, closely related to gene function and stability. The chromosome compartments are divided into transcriptionally active compartment A and repressive compartment B, and the dynamic transition between them has a decisive effect on gene expression. Topologically associating domains (TADs), as 3D spatial functional units composed of interacting gene sets within the genome, are defined by CTCF proteins and cohesin proteins at their boundaries, laying the foundation for DNA recombination and replication. Chromatin loops are circular connections between distant chromatin, playing a key role in gene activation and fine regulation (Kumar et al., 2021). The study of R-loops and 3D genome regulation provides us with a new perspective to understand epigenetics, helping us to gain a deeper understanding of the complexity of gene expression and its role in health and disease.

In the field of Fragile X Syndrome (FXS) research, the in-depth exploration of R-loops and three-dimensional genome structures has opened new research paths for pathological mechanisms that traditional epigenetic theories struggle to fully explain. In terms of R-loop research, as early as 2014, studies proposed that the CGG-expanded FMR1 gene forms a special R-loop during transcription, where the nascent RNA hybridizes with its unzipped DNA template, potentially inducing epigenetic silencing as a structural block or nucleosome-like entity (Colak et al., 2014; Groh et al., 2014). The latest literature reports that R-loop formation is a key step in using 5i or dCas9 to conditionally induce the contraction of the CGG repeat sequence in the FMR1 gene, thereby reactivating the FMR1 gene and restoring the expression of FMRP protein. This research suggests that R-loops formed under specific conditions can, on the one hand, promote DNA demethylation, thereby further enhancing the formation of R-loops through positive feedback; on the other hand, R-loops can trigger endogenous DNA repair mechanisms, especially through the MSH2/Mismatch Repair (MMR) pathway, to correct the abnormal CGG repeat sequence length in FXS cells, activate the FMR1 gene, and restore the expression of FMRP protein, thus forming a virtuous cycle. These findings confirm the dynamic role of R-loops in the pathogenesis of FXS (Lee et al., 2023); Regarding 3D genome regulation, the latest research has found that in various cell types of FXS patients, the expansion of the CGG sequence triggers the formation of large-scale H3K9me3 regions around the autosomes and the FMR1 gene, leading to significant adjustments in chromatin structure, accompanied by severe structural disorder of topologically associating domains (TADs) and chromatin loops. This disorder not only affects the normal activity of long synaptic genes but also leads to double-strand DNA breaks caused by replication stress and progressive somatic instability in short tandem repeat sequences (STRs). It is worth noting that correcting the CGG sequence with gene editing technologies such as CRISPR can reverse the above phenomena and restore the normal structure and function of chromatin (Malachowski et al., 2023). These findings further emphasize the epigenetic role of R-loops and three-dimensional genome structures in FXS, reveal the complexity of epigenetic regulation in FXS, and provide a new theoretical basis for future therapeutic strategies.

## 7 Discussion

**FIGURE 3:**
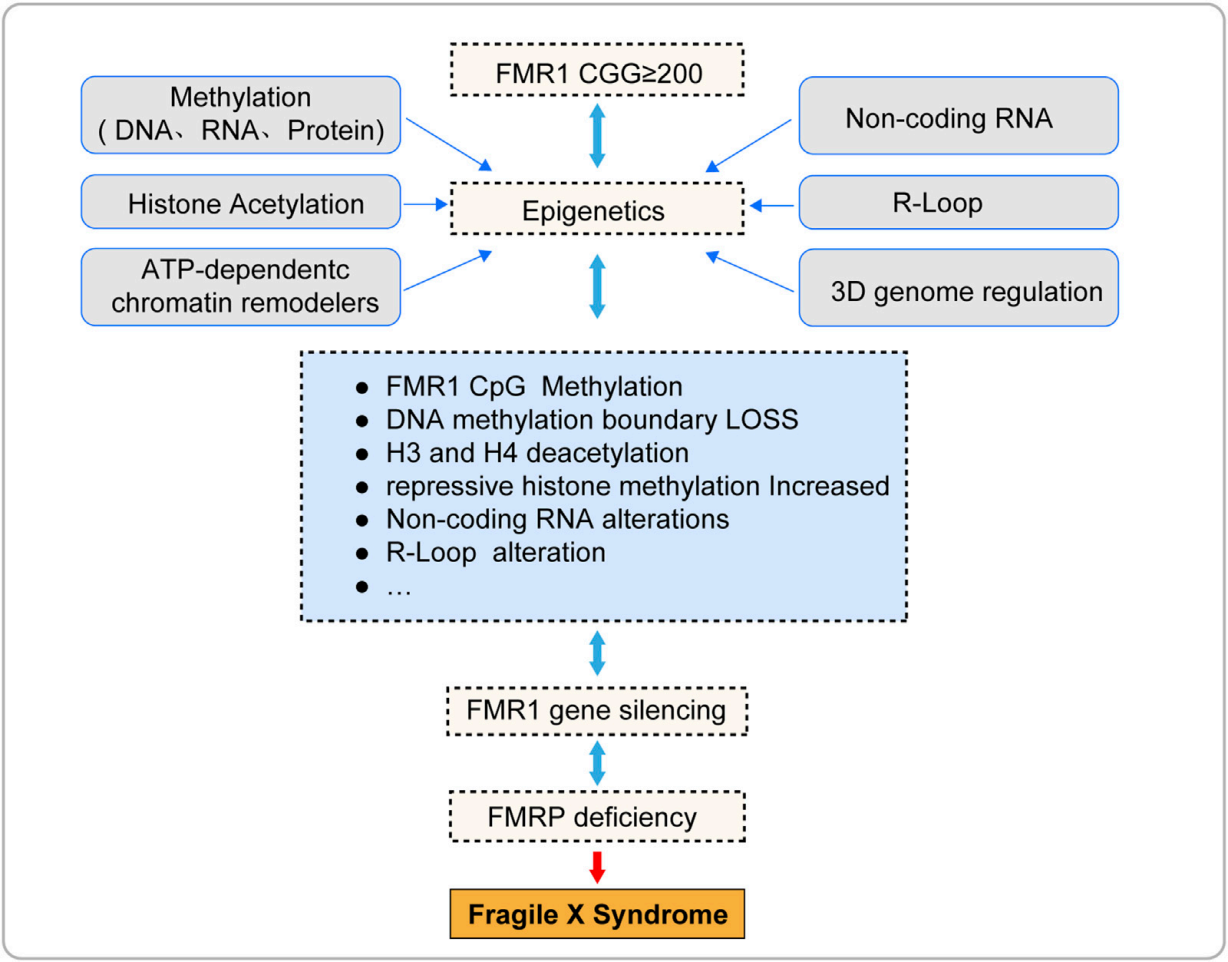
The pathogenesis of fragile X syndrome.

Epigenetic silencing of the FMR1 gene is the core pathological mechanism of Fragile X Syndrome (FXS) (Figure 3; Table 1), and its reversibility offers potential for treatment of FXS. This article reviews the current progress in epigenetic research on FXS, aiming to promote the application of epigenetics in FXS research and treatment. However, due to the complexity and interdependence of multiple steps in epigenetic processes, the precise localization of epigenetic marks during the transcription of the FMR1 gene remains a challenge, and the mechanism of FMR1 gene inactivation during development is not yet fully understood. To fully explore the potential for FXS treatment, future research needs to delve into the epigenetic mechanisms behind the silencing of the FMR1 gene, including the interactions between these epigenetic marks. This will help us to more comprehensively understand the complexity of FMR1 gene silencing and provide a solid scientific foundation for the development of effective therapeutic strategies capable of reversing these silencing marks.

**TABLE 1:** FMR1 Gene’s overview of known epigenetic modifications.

Epigenetics			Findings in FXS	References
Type	Classification	Components		
Methylation	DNA Methylation	N6 position of adenine N7 position of guanine C5 position of cytosine	• The DNA methylation boundary, located approximately 650–800 nucleotides upstream of the FMR1 gene exon 1, is lost.Loss of CpG methylation in the far 5′-upstream FMR1 region • Increased levels of 5 mC correspond to reciprocal changes in 5 hmC within the FMR1 gene body and its proximal flanking regions • increased methylation of the FMR1 intron 1 sites, specifically CpG10–12 …	Naumann et al. (2010), Naumann et al. (2014) Brasa et al. (2016) Godler et al. (2023)
	RNA Methylation	m6A	• the m6A landscape can be affected by FMRP in mouse cortex • some m6A-marked mRNA is the target of FMRP • the stability of m6A-marked mRNAs is regulated by FMRP	Zhang et al. (2018); Edens et al. (2019)
		m5C	• establishment and removal of m5C methylation modifications maybe regulated by FMRP	Yang et al. (2022); Chen et al. (2023)
	Protein Methylation	arginine \llysine residues of signaling pathway proteins or histones	• elevated H3K9Me3 and H4K20Me3 coincident with the FMR1 gene repeat • elevated H3K9Me2和H3K27Me3 on exon 1 or intron 1	Kumari and Usdin (2010)
Histone Acetylation	N/A	histone tails	• hypoacetylated histones at 5′end of the FMR1 gene	Chiurazzi et al. (1998, 1999); Coffee et al. (1999)
Chromatin Remodeling	SWI/SNF	cBAF PBAF ncBAF	• The subunit Brm of the SWI/SNF complex can interact with the methylated FMR1 gene through MeCP2. • The expression of subunits from the remodeling factor families, including Arid1, CHD, and Smarca, is altered in the FXS model.	Moffat et al. (2022); Liu et al. (2023); Muhammad et al. (2023)
	ISWI	NURF、CHRAC、ACF		
	INO80	INO80、SWR1		
	CHD	CHD1 NuRD		
non-coding RNA	small RNA	siRNA 、miRNA	• siRNA-mediated heterochromatinization may be the cause of FXS • many miRNA were altered in fmr1 knockout mice • FMR4 is silenced in FXS patients • The expression or function of FMR5 and FMR6 may change in FXS	Jin et al. (2004); Hecht et al. (2017); Shitik et al. (2020); Huang et al. (2019)
	long non-coding RNA	lncRNA、microRNA		
others	R-LOOP	N/A	• R-loop may act as a structural block or nucleosome analogy to induce epigenetic Silencing in FXS • R-loop formation is a key step in the conditional treatment of Fragile X Syndrome (FXS) models using 5i or dCas9.	Colak et al. (2014); Groh et al. (2014) Lee et al. (2023)
	3D genome regulation	chromosome territory	• TADs and chromatin loops exhibit severe structural disarray in FXS.	Malachowski et al. (2023)
		chromosomal compartment		
		TAD		
		chromatin loop		
